# Exploring carer resilience in the context of dementia: a meta-synthesis

**DOI:** 10.1186/s12877-022-03516-3

**Published:** 2022-10-20

**Authors:** Eun Young Kim, Sung Ok Chang

**Affiliations:** 1 Department of Nursing, Soonchunhyang University, Cheonan, Republic of Korea; 2 College of Nursing, and BK21 FOUR R&E Center for Learning Health Systems, Korea University, 145, Anam-ro, Seongbuk-gu, 02841 Seoul, Republic of Korea

**Keywords:** Dementia, Caregivers, Psychological resilience, Qualitative Research, Systematic Review

## Abstract

**Aims:**

The aim of this literature review is to integrate the results of qualitative research on the resilience experiences of family carers of people with dementia (PWD).

**Design:**

A qualitative meta-synthesis study was conducted.

**Methods:**

The meta-ethnography method of Noblit and Hare (1988) was used. We searched five electronic bibliographic databases (PubMed, EMBASE, CINAHL, PsycINFO and Web of Science) using the keywords “caregivers”, “family caregivers”, “spouse caregivers”, “qualitative research”, “resilience, psychological” and “dementia.” The inclusion criteria of the literature search found studies that explored the resilience experience of family carers of PWD, were qualitative, were published in English, and had participants 18 years of age or older.

**Results:**

Eleven studies, 1 from Australia, 4 from USA and 6 from UK, were included in the analysis. Through the process, three themes emerged: ‘Seeing the life of a carer as one’s duty’, ‘Setting boundaries in life’, ‘Moving forward toward a developing life.’ These themes illustrated how family carers of PWD overcome the adversities they encounter.

**Conclusion:**

This meta-synthesis showed how family carers of PWD adapt to and overcome the difficult situations they are confronted with as carers. This review suggests an important direction for enhancing the resilience of family carers of PWD.

**Supplementary Information:**

The online version contains supplementary material available at 10.1186/s12877-022-03516-3.

## Introduction

More than 55 million people worldwide have dementia, with nearly 10 million new cases occurring each year [1]. With the population of older adults steadily increasing, the importance of dementia, considered to be one of the most significant risk factors for aging populations [2], can only be expected to become more prominent in the future. The majority (80%) of people with dementia (PWD) are cared for at home by a family member or friend [3]. Each year, more than 16 million Americans provide more than 17 billion hours of unpaid care for family and friends living with dementia [3]. Due to living with a significant burden of care, family carers suffer from physical problems such as cardiovascular disease and musculoskeletal symptoms [4, 5] and mental problems such as stress, depression, and anxiety due to care [4, 6, 7]. This burden is negatively correlated with the quality of life of carers [8], and, ultimately, the quality of care provided to PWD [9]. In addition, it worsens people’s physical problems and causes serious social problems, such as abuse of PWD [10]. Therefore, we need to pay attention to the burdens of family carers of PWD, and research on protection strategies that can buffer the negative effects of those burdens is needed.

Resilience, an important concept describing protection strategies in carer research, has been defined in various ways. This concept refers to the ability of carers to adapt to the physical and psychological requirements of their role [11] and to their ability to recover from a crisis situation [12]. In addition, it refers to an individual's distinctive abilities and characteristics as they cope with adversity [13, 14], the dynamic process of positively adapting through adversity [15, 16], and the personal growth engendered by overcoming adversity that leads to significant development as a carer [17, 18]. In some previous studies, family carers of PWD were found to have high satisfaction with their caring roles and to have positive experiences despite the difficult situation, characteristics which were found to be related to resilience [19, 20]. As the severity of dementia increases, the amount of care that carers need to provide increases, and the burden on them increases [21]. However, according to a previous study, the resilience of carers caring for PWD with high severity dementia was higher than that of carers caring for PWD with low severity dementia [22]. These results indicate that resilience does not simply mean the strength to overcome difficult situations, but also includes the meaning of taking a positive approach and growing as a way to help overcome them.

The resilience of carers of PWD has been continuously studied. Their resilience has been shown to increase both their physical and mental well-being [20] while reducing their anxiety and depression and facilitating coping [23]. Recently, studies on the resilience of family carers caring for PWD have been increasing, but studies that can lead to a deeper understanding of resilience by integrating previous study results have been limited. Moreover, there are some practical limits to understanding the resilience experiences of family carers of PWD reported in previous studies due to the differences in study design and results. Therefore, this literature review integrates the results of various qualitative studies on the resilience of family carers of PWD to formulate a new interpretation and reveal comprehensive aspects of the phenomenon. This can suggest future research directions and provide basic information for interventional research that can help reduce the difficulties of carers and improve the quality of care they provide to PWD. Ultimately, it will help improve the health and quality of life of PWD.

Qualitative meta-synthesis is a method of synthesising and analysing individual qualitative research, one that is used to derive more accumulated knowledge, expand on that knowledge and produce new interpretations from the results of research areas and phenomena suggested by existing studies [24, 25]. In addition, it can enable more specific suggestions for future studies [24, 25].

To date, systematic reviews of the resilience of family carers of PWD have been conducted in various ways [19, 26, 27]; however, a qualitative meta-synthesis integrating various perspectives of the qualitative research that analysed the resilience experiences of these family carers has not been attempted. Meta-synthesis methods have the potential to integrate different perspectives by synthesising different qualitative results, generating higher-order questions, and reducing duplication of research [24, 25]. A meta-ethnographic approach has been used in the synthesis of a variety of qualitative studies, particularly those related to chronic diseases and the experiences of carers [28, 29]. Through this approach, a conceptual model that goes beyond a simple set of results can be presented, providing directions and basic data for future resilience-related research on family carers who care for PWD.

### Aim

The purpose of this literature review is to comprehensively understand in-depth the existing qualitative studies on the resilience experiences of family carers of PWD by conducting a systematic review and qualitative synthesis.

The study’s research questions were:

“What experiences do family carers of PWD have in coping with the difficult situations they face?”

“What are the adaptive capacities that enable family carers to adapt to their situation?”

### Design

This literature review utilises a qualitative meta-synthesis design that integrates and synthesises qualitative research results exploring the experiences of family carers’ resilience in PWD. From the existing qualitative meta-synthesis research methods, the seven phases of Nolit and Hare’s meta-ethnography [30] were applied. This literature review study was conducted in compliance with the guidelines of Enhancing transparency in reporting the synthesis of qualitative research statement (ENTREQ) [31].

The study was registered in the international prospective register of systematic reviews (PROSPERO: CRD42021278764).

### Literature search

A literature search was conducted in November 2021. Five electronic bibliographic databases (PubMed, EMBASE, CINAHL, PsycINFO and Web of Science) were used to conduct a literature search. The search terms were selected from the MeSH term list ("caregivers", “family caregivers”, “spouse caregivers”, "qualitative research", "resilience, psychological" and "dementia") and were used in the search in different combinations with the Boolean operators "AND" and "OR". Publication years were not limited for the comprehensive literature searches. The quality of the literature search was confirmed through consultation with a professional librarian.

The inclusion criteria applied to the research search were as follows: (a) studies aimed at examining the resilience experiences of family carers of PWD, (b) studies using qualitative research methodology, (c) studies published in peer-reviewed journals, (d) studies published in English and (e) studies that included participants older than 18 years. The exclusion criteria were as follows: (a) studies not suitable for the purpose of this study, (b) studies not published in English, (c) studies in which qualitative methods were not used, (d) studies with children or adolescents and (e) systematic reviews, meta-syntheses, and secondary analyses.

**Fig. 1:**
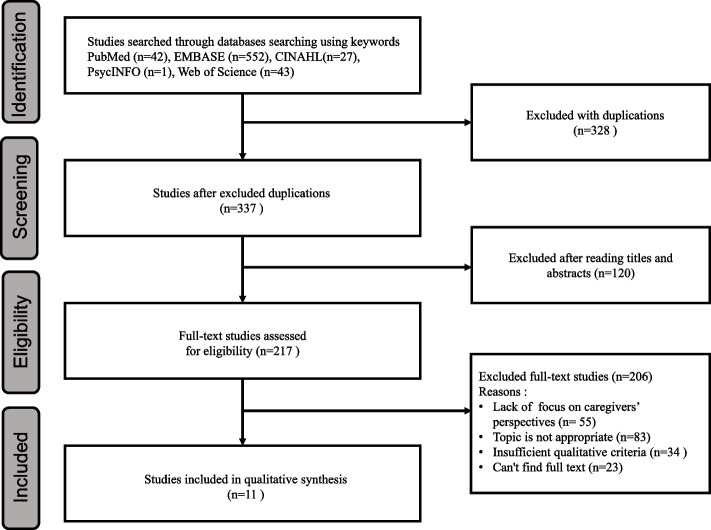
Flow Chart of Systematic Review of Literature Selection Process

A flow chart of systematic review of literature selection process is presented in Fig. 1. We identified 665 studies and excluded 328 duplicates. After examining the study titles and abstracts to ensure that they met the inclusion criteria, we excluded 120 studies. After scrutinising the full texts of the remaining 217 studies to assess their eligibility according to the inclusion criteria, we eliminated 206 studies. Eleven studies were chosen for the final review (see Supplementary Material S1 and S2 for search strategies & final literature selected for review).

### Quality appraisal

To evaluate the quality of the 11 included studies, the Critical Appraisal Skills Programme (CASP) qualitative checklist, consisting of 10 questions, was used to evaluate each study’s reliability, truthfulness and rigor [32]. Each of the two researchers evaluated each study using the CASP checklist, and any disagreements between the evaluations were resolved through discussion. As a result of the evaluation, four studies met 70% of the CASP criteria, four met 80% of the CASP criteria and three met 90% of the CASP criteria; no studies were excluded. The CASP results are presented in Supplementary Material S3.

### Data extraction

The two researchers read each study intensively and repeatedly to understand and familiarise themselves with the studies’ details. The two researchers independently extracted data that were considered meaningful and relevant to the resilience experiences of family carers of PWD, including author details, participant characteristics, research methods and citations, and organised them using a customised format in Microsoft Excel. The data were extracted into first-order constructs and second-order constructs. First-order constructs refer to the participants' interpretations in original studies, second-order constructs refer to the original researchers' interpretations and third-order constructs refer to the researchers' new interpretations concerning the first- and second-order constructs [24, 25].

### Data synthesis

Employing the meta-ethnography method, the researchers continuously contrasted and compared the first- and second-order constructs. To clarify the concept, key concepts were extracted from the first- and second-order constructs, and the third-order constructs, which are regarded as the main themes, were derived through a process of abstraction by the researchers [33]. During the process of analysis and synthesis, the researchers discussed and resolved differences in interpretation that arose due to differences in academic and clinical backgrounds.

### Ethical approval

Because this research is a literature review study, ethical approval and informed consent were not required.

## Results

Analysis was undertaken of 11 studies with a total of 221 participants (Table 1). One study was conducted in Australia, six in the United Kingdom and four in the United States. Of the participants, 76% were female. Participant age varied from those in their 20 s to those in their 80 s, but the average age was 67.6. In addition, 117 (53%) were spouses, 85 were children (38%), 6 were grandchildren (3%), 6 were significant others (3%), 4 were siblings (2%) and 3 were friends (1%). Four of the studies provided participant employment status, and 44.3% of those participants were employed full-time or part-time. The duration of care varied from less than 1 year to 13 years. As a result of the synthesis of 11 qualitative literature analyses, 3 themes, and 6 sub-themes were derived (Table 2).

**Table 1:** Summary of the included studies

Article. No	Author, year/Country	Sample size (M:F)	Carer age range(years)	Type of carer (n)	Employment	Caring period range (years)	Research aim	Research design	Data collection methods	Data analysis	Percentage that meets CASP
A1	O’Dwyer et al., 2013 / Australia	9 (4:5)	25–82 (mean: 58.3)	Daughter (3), Spouse(4), Son-in-law (1), Grandson (1)	Part-time: 3 Unemployed: 5 Job seeker:1	0.5–11	To conduct an initial exploration of carers’ experiences of suicidality and identify factors associated with risk and resilience, which could be used to guide further research	A descriptive qualitative study	In-depth interview	Thematic analysis	80%
A2	Donnellan et al., 2015/UK	20 (7:13)	62–89 (mean: 76.0)	Spouse (20)	Not presented	2–10	To assess whether spousal dementia carers can achieve resilience and to highlight which assets and resources they draw on to facilitate or hinder resilience, using an ecological framework	A qualitative study	In-depth interview	A grounded theory	80%
A3	Donnellan et al., 2017 / UK	23(7:16)	62–89 (mean: 75.1)	Spouse (23)	Not presented	2–10	to explore social support as a key component of resilience to identify the availability, function and perceived functional aspects of support provided to older spousal dementia carers	A qualitative study	In-depth interview	A grounded theory	90%
A4	Roberts et al., 2018/USA	33(4:29)	39–83 (mean: 65.8)	Daughter, son, daughter-in-law, son-in-law: 18 Wife: 12 Husband: 2 Sibling:1	Not presented	Not presented	To address this major public health challenge through the lens of caregiver resilience and caregiver respite programming	A mixed-method study	Face-to-face interview	Thematic analysis	70%
A5	Jones et al., 2019/UK	13(Not presented)	40–81 (median: 61)	Wife: 6 Son:1 Housemate:1 Daughter:2 Husband:2 Daughter in law:1	Part-time: 2 Retired: 8 Full time:2 Not working: 1	Not presented	(a) explore discrepancies and congruency between definitions of resilience in the academic literature and carers own conceptualisations; (b) assess differences and similarities in conceptualisations of resilience between carers with high, medium and low resilience scores; (c) compare carers’ perceived level of resilience with the level of resilience when measured on a standardized tool	A cross-sectional qualitative study	Semi-stuctured interviews	Qualitative analysis	80%
A6	Donnellan et al., 2018/UK	13(4:9)	65–85 (mean: 75.4)	Spouse: 13	Not presented	3–13	To examine trajectories of resilience and which assets and resources are associated with resilience and care status transitions in spousal dementia carers	A qualitative longitudinal study	In-depth interview	A grounded theory	90%
A7	Han et al., 2019/USA	39(9:30)	Mean:62	Adult child:82.1% Spouse/partner:7.7% Niece:5.1% Friend: 5.1%	Full or part time job: 18 Not employed: 21	6 months or less—3 years or more	To identify challenges, possible solutions that are resources for resilience, and expected consequences from the perspective of family caregivers of hospice patients with dementia	A theory-driven, deductive content analysis study of secondary data obtained from a clinical trial	Individual interview	Content analysis	70%
A8	Conway et al., 2020/UK	12(Not presented	Not presented	Spouse/partner: 12	Not presented	3 month—6	To explore what resilience means in the context of couplehood in dementia, how dyads experience a shared sense of resilience, how they develop and maintain resilience and how this impacts upon their relationship	A qualitative study	In-depth interview	Constructive grounded theory	90%
A9	Jensen et al., 2020/USA	19(4:15)	20 s-80 s	Child:7 Grandchild: 5 Spouse/significant other: 1 Sibling: 2 Other family member:3 Friend: 1	Not presented	Not presented	to identify characteristics of resilience using surveys in 50 bereaved caregivers for persons with dementia who lost their care recipient in the past 6 month	A qualitative descriptive study	Individual interview	Content analysis	80%
A10	Donnellan et al., 2021/UK	13(2:11	47–81 (mean: 66.0)	Adult daughters: 6 Spouse: 7	Not presented	1–9	To identify the factors that facilitate or hinder resilience in spousal and adult daughter carers, and whether these factors can be mapped on to ecological resilience framework	A qualitative study	Semi-structured interview	Constructive grounded theory	90%
A11	Liu et al., 2021/USA	27(6:21)	50–89 (mean: 69.0)	Spouse: 46% Adult Children: 50% Sibling:4%	Full-time or part-time employed: 9 Retired or unemployed: 18	Mean: 2.48	To investigate the resilience of a growing but largely underserved and understudied population—Chinese American dementia caregivers	A qualitative study	Semi-structured interview	Hybrid grounded theory model	70%

*F* Female, *M* Male, *CASP* Critical Appraisal Skills Programme checklist

**Table 2:** Synthesized themes of resilience of family cares of people with dementia

Key concepts from first-and second order constructs	Sub-themes	Synthesized themes
Past good memories ^A1, A3, A5, A8, A9^ Affection with family ^A2, A3, A4, A8, A10^ Understanding about life of patients ^A2, A7, A8, A10^ Sharing experiences ^A2, A3, A8, A10^ Building trust relationships with patients ^A2, A4, A7, A8, A10^	1. Building a sense of bonding based on life with people with dementia	I. Seeing life as a carer as one's duty
Accepting the current situation ^A1, A2, A4, A5, A7, A8, A10^ Awareness of the finiteness of the situation ^A1, A2, A5, A8, A9^ Expressing/controlling their own emotions ^A1, A2, A3, A4, A5, A7^ Perceiving the value of their life ^A2, A3, A4, A5, A7, A8^ Taking the situation positively ^A1, A2, A5, A6, A8, A9, A10^	2. Acknowledging their life as a carer	
Building a sense of unity from the same carer community ^A1, A2, A3, A5, A6, A7, A8, A11^ Getting help from their family ^A1, A2, A3, A6, A7, A8, A9, A10^ Interacting with the community (social support) ^A1, A2, A3, A5, A6, A7, A8, A10, A11^ Communicating with friends and neighbors ^A1, A2, A3, A5, A6, A7, A8, A9, A10^ Receiving help from experts ^A2, A7, A8^	3. Finding stable life through help from supportive relationships	II. Setting boundaries in life
Having personal time ^A1, A2, A4, A5, A6, A7, A9, A11^ Striving for self-development ^A1, A2, A7, A11^ Trying to maintain one's identity ^A1, A2, A5, A6, A7, A9, A11^ Separating themselves from patient care ^A1, A2, A4, A5, A6, A7, A9, A10, A11^ Focusing on the present ^A2, A4, A5, A10, A11^ Recognizing the importance of rest ^A1, A2, A4, A5, A6, A10, A11^	4. Rediscovering independent life	
Staying work life ^A1, A4, A6, A8, A10^ Doing one’s role ^A4, A5, A10, A11^ Taking one’s responsibility ^A4, A5, A10, A11^ Managing their routine activities ^A1, A2, A5, A10, A11^ Financial reward ^A2, A5, A6, A7, A8^	5. Maintaining sociality	III. Moving forward towards a developing life
Learning professional knowledge ^A1, A2, A5, A7^ Building confidence ^A1, A2, A4, A5, A7^ Be active in life (as a carer) ^A1, A2, A4, A5, A7^ Developing skills and insights ^A1, A2, A4, A5, A7^	6. Developing professionalism in the role of carers	

*PWD* People with dementia

### I. Seeing life as a carer as one’s duty

The family carers developed bonds with the PWD based on their lives with them, and they acknowledged and accepted their roles as carers. They considered life as a carer to be a duty and tried to fulfill that duty. This sense of duty became the driving force behind their work as carers, and this became the basis for their overcoming difficult situations.

#### Building a sense of bonding based on life with PWD

Good past memories (A1,3,5,8,9) made with PWD and the affections shared with family members (A2,3,4,7,10) acted as positive forces when family carers had to cope with difficult situations. In their efforts to understand PWD and their lives (A2,7,8,10), family carers found meaning in sharing their experiences (A2,3,8,10) and forming trusting relationships with their PWD (A2,4,7,8,10), eventually building a sense of bonding. The formation of a sense of bonding provided justification for their roles as carers and became the basis for overcoming difficult situations.

*We’ve been together nearly 50 years. Would I feel like this if I’d only been together 7, 8, 10?......We’re comfortable. We've been together for so long, so it couldn't be more comfortable. (A8).*

*My dad became acutely ill with aspirate pneumonia, so that we had 5 days as a family to come together. We kept a vigil so that he was never left alone, and most of the time there was more than one of us there 24/7. We had time together individually with him and as a family. Many memories and stories were shared in the middle of the night—I cherish that time (A9).*

#### Acknowledging their life as a carer

The participants accepted their current situations, (A1,2,4,5,7,8,10) rather than complaining about the situation, recognising that it was a finite one with an eventual end (A1,2,5,8,9). They tried to express their feelings without hiding them (A1,2,3,4,5,7), acknowledging the importance and value of their work (A2,3,4,5,7,8) and of themselves. They perceived their situation positively and wanted to view their lives in a positive way (A1,2,5,6,8,9,10).

*I’m positive. I laugh and I sing and she laughs and I act gently in the house. I’ve even talked to one of my neighbours about my singing, and she said [Mr Go.] it’s a good thing we’ve got a detached house. I sing at the top of my voice (A2).*

*‘Doing what you can, if there's something you can't do, don't do it or do it differently. Look for the positives and don't beat yourself up (A5).*

### II. Setting boundaries in life

Carers, who live a life in which the boundaries between their own lives and the lives of PWD are ambiguous, set the boundaries of their lives. Rather than shouldering the difficult burdens of their lives alone, they actively sought supportive relationships. They wanted to focus on their own lives and lead independent lives.

#### Finding a stable life through help from supportive relationships

They realised that it would be more helpful to rely on different forms of support than to tackle their difficult situations alone. They found help by joining a local community or self-help group of people in the same situation, gaining solace from them, and forming a sense of unity (A1,2,3,4,6,7,8,11). They also received help from their families, who directly assisted with the act of caring while providing them psychological stability (A1,2,3,6,7,8,9,10). In addition, they sought social support from important acquaintances around them and received help (A1,2,3,5,6,7,8.10,11) While forming new social relationships, they maintained good social relations with friends and neighbors, from whom they received help (A1,2,3,5,6,7,8,9,10). They also got help from experts who can provide professional advice when they needed it (A2,7,8). They did not hesitate to seek help and actively sought out the help available to them, and supportive relationships provided them with stability.

*I [got] involved somehow with advocacy [for other carers]... I quickly became empowered...and then I was actually advocating for other people, so that was one way that I coped (A1).*

*Coming here(self-help group) has helped me because the people that come here are in the same position as I am. Some of them have been in it longer than me, so I can use their experience and I can relate to what they’re saying (A2).*

*‘The carer group is a godsend because sometimes you’ve just got to dump and you can do it there. It makes me feel better because I know I’m not alone. Every other one of those wives is going through what I’m going through. It’s the neatest, tiredest looking group of women I’ve ever seen. We have days when we laugh and cry; it’s like this little amount of light. Without the groups, I wouldn’t have made it’ (A4).*

#### Rediscovering an independent life

By building their own lives and domains separate from their roles as carers, they were able to develop the strength to overcome the crisis of dealing with dementia. They tried to relieve their stress by spending personal time away from caring (A1,2,4,5,6,7,8,9,11). They focused on self-development (A1,2,7,11) and made efforts to maintain their individual identities (A1,2,5,6,7,9,11). To do this, the carers focused on time (A2,4,5,10,11) and tried to feel separated from their time caring for the PWD (A1,2,4,5,6,7,9,10,11), realising the importance of rest and trying to secure the time necessary for sufficient rest (A1,2,4,5,6,10,11). Creating temporal space for themselves in these ways was recognised as an effective method to cope with their difficult situations.

*I love getting up in the morning at 5 am, going for my hour’s walk, and that’s my time ... that’s my “clear my head” time. If I didn’t have that right now, I think things would be different, but it’s just giving me that little bit more strength to think straight (A1).*

*I’m getting a respite on weekends. I really do know the meaning of recharging my batteries now. I feel more, you know, on Monday morning right, let’s get on with the day (A2).*

*If I feel stressed, I sing loudly when taking a shower. … I like singing old songs that were popular when I was young (A11).*

### III. Moving forward toward a developing life

Carers tried not to become trapped in the work of caregiving. They sought to continue a life away from caregiving, maintaining their sociality while preserving their social positions. In addition, they tried to develop their skills as carers. Maintaining sociality and developing oneself were considered as one of the ways to hold on to their individual identities.

#### Maintaining sociality

Among the carers, there were those who had their own jobs other than as carers. They sought to maintain a social life and relationships (A1,4,6,8,10), while faithfully carrying out their assigned duties as carers (A4,5,10,11). They tried to manage their daily lives well (A1,2,5,10,11) and did their best in their responsibilities as carers (A4,5,10,11). They sometimes received financial rewards from social support services, communities of faith and other family members which were viewed as positive for handling their difficult situations (A2,5,6,7,8).

*My brother-in-law moved her (mother-in-law) in with him to start with, but he worked during the day and I was concerned about her well-being during the day. I was afraid she was just eating Twinkies and things, not being able to prepare meals. I work from home, and I thought I could take care of her and work from home, so we brought her to our house (A4).*

*When I am at work…..we’ll have a laugh about it (working). I think going to work helps me a lot (A10).*

#### Developing professionalism in the role of carers

They strived to acquire professional knowledge (A1,2,5,7), gained confidence in their work (A1,2,4,5,7), and tried to live an active life as carers (A1,2,4,5,7). They also did their best to develop skills and insights related to care (A1,2,4,5,7).

*I went on the internet, looked up what medication he should be on... and I was like a dog with a bone. We just became proactive. Within a couple of weeks I went to the Alzheimer’s [support center] and I just sort of took on board everything. But it’s 9 years later, and you’re still learning all the time (A2).*

*I insist on exercising at home every day. Before he [care receiver] wakes up, I have some time to do that. If my health is poor, how can I take care of him? (A11).*

### IV. The conceptual model of the resilience of family carers of PWD

**Fig. 2:**
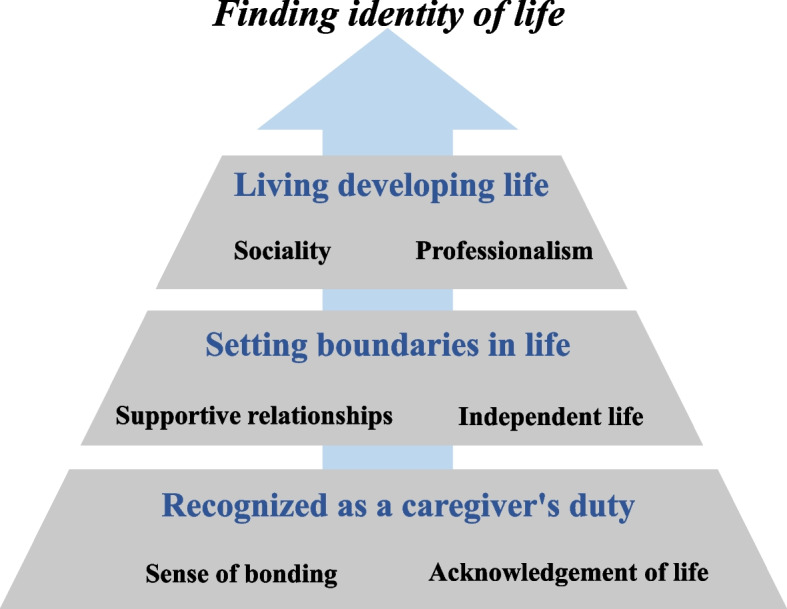
The Conceptual Model of the Resilience of Family Caregivers of People with Dementia

The resilience experience of carers of PWD can be expressed as Fig. 2. Carers recognise a sense of duty as a carer through their bond with PWD and acknowledgement of the carer's life, and this sense of duty is the basis for their lives as carers. They also set boundaries between their own lives and their role as carers by seeking help from supportive relationships and pursuing an independent life. Carers plan and live a developing life based on this clarity in their lives. This series of processes can be expressed comprehensively as rediscovering one’s identity.

## Discussion

In an aging society, family carers of PWD face physical and mental challenges from bearing the burden of care [34]. Since the difficulties of carers of PWD significantly affect the quality of care they provide as well as their own quality of life [8, 9], social attention is needed for supporting such carers as they seek ways to cope with the challenges they face. The results of this literature review can provide a comprehensive understanding of the resilience of family carers with PWD by integrating the findings of various qualitative studies. In addition, utilising the resilience experiences and characteristics of family carers of PWD, we seek to suggest the use of identified themes.

The results of this literature review study suggested that resilience is a process of adapting to a difficult situation [15, 16] and has the meaning of growth so as to develop one step at a time [17, 18], supporting the results of previous studies that define resilience. Ultimately, family carers develop resilience through this series of processes and discover their identity in life.

The informal carers or partners reported attempting to understand the person with dementia, protect that person, and adapt to the situation by living a life of symbiosis with that person [29, 35]. On the other hand, in this meta-synthesis, carers showed the will to build their own lives while acknowledging their lives as carers. In addition, it was found that carers tried to develop their lives further, rather than being isolated, and used various methods to overcome their difficult situations. Increasing carer resilience in the context their positive will is worthy of further investigation.

According to the results of this review, family carers of PWD displayed an attitude that acknowledged their life as a carer. In this process, it was revealed that they especially valued the affection and relationship of the family. These results can be supported by those of previous studies that showed the relationship with PWD and intimacy with the family had a positive effect on carers’ abilities to overcome difficulties [26]. In addition, carers worked to adapt to their situation by thinking about the value of life while remembering how important their role as a carer was. Showing similar results to this study, a study on the resilience of carers of people with hemodialysis revealed that they sought to find meaning in their lives and to accept their situation positively to cope with their condition(s) [29]. Therefore, we can provide programs that can help PWD or their families find the meaning of life and form a stronger attachment to life, along with interventions that can help them realise the value of life and view their situations positively. Such attempts may play a positive role in enhancing resilience improvement.

In addition, as a result of the literature review, it was found that carers on their own seek ways to create a boundary between their personal lives and their lives as carers and to reduce the burden of caring. Carers were not reluctant to receive help, actively seeking out organisations and methods from which they could receive help. In a previous study, it was determined that the longer the time of caregiving, the greater the burden became for family carers of PWD [28]. Providing direct and indirect support will make it possible to reduce the burden of care on the families of PWD. This can be linked to resilience building for the carer through actively seeking ways to receive help directly for reducing the burden of caring. Medical experts can help identify these family carers’ needs and help connect them with appropriate support. In a previous study that studied other care situations, carers of people with chronic diseases focused on their burden [36, 37], but the results of this review revealed that family carers of PWD tend to seek out their own identity in life. Therefore, there is a need for a strategy that respects the lives of carers of PWD and can help them lead independent lives. A variety of educational resources should be developed to support the human resources that can actually help carers, and to secure expertise in providing care.

Family carers wanted to move forward toward an evolving life, even if they were in a difficult situation. They wanted to maintain their jobs, fulfill their roles and responsibilities as carers, and sustain their daily routines. In addition, financial compensation from social support services, communities of faith and other family members was a helpful factor for them. In previous studies, family carers underwent negative psychological experiences, such as worsening mental health and increasing life stress levels, due to job instability including unemployment and turnover [38]. Therefore, the results of this meta-synthesis implies methods of reducing their burden of care, which is interpreted as having an important meaning in the resilience of family carers of PWD. Moreover, this supports the previous research that growth and development are the main attributes of resilience [39, 40]. Based on this understanding of how family carers of PWD have adapted to difficult situations, it is necessary to develop and provide various platforms so that they can develop their expertise. Their will for growth can change the paradigm for future family carers of PWD, and they can grow as a professional group with a new specialty. Such a positive movement could provide an important solution to the social difficulties related to caring for PWD.

### Limitations

This meta-synthesis has three limitations. First, there are limitations in generalizing the results of this study, because the studies for this work were from 3 westernised countries with a very similar culture base and health systems limiting translation into other different cultures and health systems.

Second, since the degree of dementia was not indicated in the literature included in this literature review, it was difficult to confirm whether the resilience experienced by the carers differed according to the degree of dementia. If future research reveals that the resilience experience of carers differs according to the degree of dementia, suggesting more situation-specific approaches to improve the resilience of carers caring for PWD would be worthwhile.

Third, it was found that family carers consisted of various family members, with the majority, 53%, being spouses. However, since different family member carers were participants in the articles reviewed, it was difficult to distinguish the characteristics of the resilience experiences of spouse carers. This demonstrates the necessity of conducting more detailed studies on the resilience of spouse carers and that of adult children carers to identify the characteristics of each. In addition, this meta-synthesis did not specify whether or not family carers lived with PWD. In order to consider the quality of life of family carers and their lives separately from their roles as carers, future studies should consider the resilience of family carers according to whether or not they live together with a person with dementia.

## Conclusion

This literature review suggested the experiences of family carers of PWD in the process of overcoming the challenges they faced in their lives as carers. While acknowledging their life as a carer, they tried to live an independent life while establishing boundaries between that life and their life as a carer, all as part of moving forward toward a developing life. Ultimately, they developed resilience by finding an identity for their lives. These results can provide direction for promoting the resilience of carers of PWD and for improving their quality of life.

Developing an understanding that carer resilience is a cornerstone for designing a positive future for both carers and PWD, is important. Our findings offer some initial approaches as to how medical professionals might facilitate and support carer resilience and ameliorate carer burden. However, measures to improve resilience should be developed at both policy and practice levels with a view to their incorporation into best practice.

## Supplementary Information

**Additional file 1: Supplementary material S1.** Search strategy. **Supplementary material S2.** Final literature selected for review. **Supplementary material S3.** Quality assessment results of included studies using the Critical Appraisal Screening Program.

## Data Availability

All data generated or analysed during this study is included in this published article.
