# Supplementary material for: Exploring carer resilience in the context of dementia: a meta-synthesis
Source: BMC Geriatr. 2022 Oct 20;22:806. doi: 10.1186/s12877-022-03516-3 (PMC9585856; doi:10.1186/s12877-022-03516-3)
Supplement: Supplementary file 1 — Additional file 1: Supplementary material S1. Search strategy. Supplementary material S2. Final literature selected for review. Supplementary material S3. Quality assessment results of included studies using the Critical Appraisal Screening Program. [file 12877_2022_3516_MOESM1_ESM.docx]

Supplementary material S1. Search strategy

| **Databases** | **Search No.** | **Query** | **Results** |
| --- | --- | --- | --- |
| **Pubmed (MeSH)** | #1 | (Caregivers[MeSH Terms] OR caregiver*[Title/Abstract] OR carer*[Title/Abstract] OR spouse[Title/Abstract] OR family[Title/Abstract]) | 960,632 |
|  | #2 | "Qualitative Research"[Mesh] OR "Qualitative research"[tiab] OR "focus group"[Title/Abstract] OR "hermeneutics"[Title/Abstract] OR "grounded theory"[Title/Abstract] OR "interview"[Title/Abstract] | 250,700 |
|  | #3 | "Resilience, Psychological"[MeSH Terms] OR "resilien*"[Title/Abstract] | 44,813 |
|  | #4 | "dementia"[MeSH Terms] OR "dementia"[All Fields] OR "dementias"[All Fields] OR "dementia s"[All Fields] OR ("alzheimer disease"[MeSH Terms] OR ("alzheimer"[All Fields] AND "disease"[All Fields]) OR "alzheimer disease"[All Fields] OR ("alzheimer s"[All Fields] AND "disease"[All Fields]) OR "alzheimer s disease"[All Fields]) | 288,456 |
|  | #5 | (((((dementia) OR (alzheimer's disease)) AND ((Caregivers[MeSH Terms] OR caregiver*[Title/Abstract] OR carer*[Title/Abstract] OR spouse[Title/Abstract] OR family[Title/Abstract]))) AND ("Qualitative Research"[Mesh] OR "Qualitative research"[tiab] OR "focus group"[Title/Abstract] OR "hermeneutics"[Title/Abstract] OR "grounded theory"[Title/Abstract] OR "interview"[Title/Abstract])) AND ("Resilience, Psychological"[MeSH Terms] OR "resilien*"[Title/Abstract])) AND ((((Caregivers[MeSH Terms] OR caregiver*[Title/Abstract] OR carer*[Title/Abstract] OR spouse[Title/Abstract] OR family[Title/Abstract])) AND ("Qualitative Research"[Mesh] OR "Qualitative research"[tiab] OR "focus group"[Title/Abstract] OR "hermeneutics"[Title/Abstract] OR "grounded theory"[Title/Abstract] OR "interview"[Title/Abstract])) AND ("Resilience, Psychological"[MeSH Terms] OR "resilien*"[Title/Abstract])) | 45 |
|  | #6 | Filters applied: Humans, English | **42** |
| **EMBASE (emtree)** | #1 | 'caregiver'/exp OR 'caregiver' OR 'care giver'/exp OR 'care giver' OR 'caregivers'/exp OR 'caregivers' OR 'carer'/exp OR 'carer' OR 'carers'/exp OR 'carers' OR 'family caregiver'/exp OR 'family caregiver' OR 'family caregivers'/exp OR 'family caregivers' | 146,648 |
|  | #2 | 'psychological resilience'/exp OR 'psychological resilience' OR 'emotional resilience'/exp OR 'emotional resilience' OR 'personal resilience'/exp OR 'personal resilience' OR 'personal stress resilience'/exp OR 'personal stress resilience' OR 'psychological stress resilience'/exp OR 'psychological stress resilience' OR 'resilience'/exp OR 'resilience' OR 'psychological' OR 'stress resilience (person)'/exp OR 'stress resilience (person)' | 964,508 |
|  | #3 | ('qualitative research'/exp OR 'qualitative research' OR 'hermeneutics'/exp OR 'hermeneutics' OR 'qualitative studies'/exp OR 'qualitative studies' OR 'qualitative study'/exp OR 'qualitative study') OR 'experience*' OR ('perception'/exp OR 'perception' | 2,273,897 |
|  | #4 | 'dementia'/exp OR 'dementia' OR 'amentia'/exp OR 'amentia' OR 'demention'/exp OR 'demention' | 431,899 |
|  | #5 | ('caregiver'/exp OR 'caregiver' OR 'care giver'/exp OR 'care giver' OR 'caregivers'/exp OR 'caregivers' OR 'carer'/exp OR 'carer' OR 'carers'/exp OR 'carers' OR 'family caregiver'/exp OR 'family caregiver' OR 'family caregivers'/exp OR 'family caregivers') AND ('psychological resilience'/exp OR 'psychological resilience' OR 'emotional resilience'/exp OR 'emotional resilience' OR 'personal resilience'/exp OR 'personal resilience' OR 'personal stress resilience'/exp OR 'personal stress resilience' OR 'psychological stress resilience'/exp OR 'psychological stress resilience' OR 'resilience'/exp OR 'resilience' OR 'psychological' OR 'stress resilience (person)'/exp OR 'stress resilience (person)') AND (('qualitative research'/exp OR 'qualitative research' OR 'hermeneutics'/exp OR 'hermeneutics' OR 'qualitative studies'/exp OR 'qualitative studies' OR 'qualitative study'/exp OR 'qualitative study') OR 'experience*' OR ('perception'/exp OR 'perception')) AND ('dementia'/exp OR 'dementia' OR 'amentia'/exp OR 'amentia' OR 'demention'/exp OR 'demention') | 1461 |
|  | #6 | Filters applied: Human, English | **552** |
| **CINAHL** | #1 | MM “dementia” OR “alzheimers” OR “cognitive impairment” | 30,685 |
|  | #2 | AB “dementia” OR “alzheimers” OR ”cognitive impairment” | 73,276 |
|  | #3 | MM “caregivers” OR “family members” OR “relatives” OR “informal caregivers” | 23,567 |
|  | #4 | AB “caregivers” OR “family members” OR “relatives” OR “informal caregivers” | 212,914 |
|  | #5 | MM “resilience” OR “resiliency” OR “resilient” | 534 |
|  | #6 | AB “resilience” OR “resiliency” OR “resilient” | 14,385 |
|  | #7 | MM “qualitative research” OR “qualitative study” OR “qualitative methods” OR “interview” | 171,298 |
|  | #8 | AB “qualitative research” OR “qualitative study” OR “qualitative methods” OR “interview” | 224,061 |
|  | #9 | #1 OR #2 | 85,037 |
|  | #10 | #3 OR #4 | 222,836 |
|  | #11 | #5 OR #6 | 14,549 |
|  | #12 | #7 OR #8 | 224,121 |
|  | #13 | #9 AND #10 AND #11 AND #12 | **27** |
| **PsycINFO** | #1 | TI ( dementia or memory loss or alzheimers ) OR AB ( dementia or memory loss or alzheimers ) | 303 |
|  | #2 | TI ( caregivers or family members or relatives or informal caregivers ) OR AB ( caregivers or family members or relatives or informal caregivers ) | 5,384 |
|  | #3 | TI ( resilience or resiliency or resilient) OR AB ( resilience or resiliency or resilient ) | 1,214 |
|  | #4 | TI ( qualitative research or qualitative study or qualitative methods or interview ) OR AB ( qualitative research or qualitative study or qualitative methods or interview ) | 3,862 |
|  | #5 | #1 AND #2 AND #3 AND #4 | **1** |
| **Web of Science** | #1 | (((TS=("dementia*")) AND TS=("caregiver*" OR "carer*" OR "family" OR "spouse*")) AND TS=("resilien*")) AND TS=("qualitative research" OR "qualitative study" OR "interview" OR "focus group" OR "grounded theory" OR "phenomenology") | **43** |

Supplementary material S2. Final literature selected for review

A1. O'Dwyer S, Moyle W, van Wyk S. Suicidal ideation and resilience in family carers of people with dementia: a pilot qualitative study. Aging Ment Health. 2013;17(6):753-60. https://doi.org/10.1080/13607863.2013.789001

A2. Donnellan WJ, Bennett KM, Soulsby LK. What are the factors that facilitate or hinder resilience in older spousal dementia carers? A qualitative study. Aging Ment Health. 2015;19(10):932-39. https://doi.org/10.1080/13607863.2014.977771

A3. Donnellan WJ, Bennett KM, Soulsby LK. Family close but friends closer: exploring social support and resilience in older spousal dementia carers. Aging Ment Health. 2017;21(11):1222-28. https://doi.org/10.1080/13607863.2016.1209734

A4. Roberts E, Struckmeyer KM. The impact of respite programming on caregiver resilience in dementia care: A qualitative examination of family caregiver perspectives. Inquiry. 2018;55:46958017751507. https://doi.org/10.1177/0046958017751507

A5. Jones SM, Mioshi E, Killett A. Coping but not allowing the coping to be everything: Resilience in informal dementia care. Health Soc Care Community. 2019;27(4):e289-e97. https://doi.org/10.1111/hsc.12732

A6. Donnellan WJ, Bennett KM, Soulsby LK. How does carer resilience change over time and care status? A qualitative longitudinal study. Aging Ment Health. 2019;23(11):1510-16. https://doi.org/10.1080/13607863.2018.1503998

A7. Han S, Chi N-C, Han C, Oliver DP, Washington K, Demiri G. Adapting the resilience framework for family caregivers of hospice patients with dementia. Am J Alzheimer’s Dis Other Demen. 2019;34(6):399-411. https://doi.org/10.1177/1533317519862095.

A8. Conway L, Wolverson E, Clarke C. Shared experiences of resilience amongst couples where one partner is living with dementia-A grounded theory study. Front Med (Lausanne). 2020;7:219. https://doi.org/10.3389/fmed.2020.00219

A9. Jensen EJ, Supiano KP, Luptak M, Anderson T, Beynon C. Resilience in bereaved caregivers of persons with dementia. J Gerontol Nurs. 2020;46(1):30-6. https://doi.org/10.3928/00989134-20191022-02

A10. Donnellan WJ, Bennett KM. Watson N. "Exploring resilience in adult daughter and spousal carers of people living with dementia in North West England: an ecological approach". Qual. Ageing Older Adults. 2021;22(1):40-55. https://doi.org/10.1108/QAOA-12-2020-0063

A11. Liu J, Lou Y, Wu B, Mui ACY. "I've been always strong to conquer any suffering:" challenges and resilience of Chinese American dementia caregivers in a life course perspective. Aging Ment Health 2021;25(9):1716-24. https://doi.org/10.1080/13607863.2020.1793900

Supplementary material S3. Quality assessment results of included studies using the Critical Appraisal Screening Program

| Article No. | A1 | A2 | A3 | A4 | A5 | A6 | A7 | A8 | A9 | A10 | A11 |
| --- | --- | --- | --- | --- | --- | --- | --- | --- | --- | --- | --- |
| Items |  |  |  |  |  |  |  |  |  |  |  |
| Item 1. Was there a clear statement of the aims of the research? | Y | Y | Y | Y | N | Y | Y | Y | Y | Y | Y |
| Item 2. Is a qualitative methodology appropriate? | Y | Y | Y | Y | Y | Y | Y | Y | Y | Y | Y |
| Item 3. Was the research design appropriate to address the aims of the research? | Y | Y | Y | Y | Y | Y | Y | Y | Y | Y | Y |
| Item 4. Was the recruitment strategy appropriate to the aims of the research? | N | N | Y | N | Y | Y | N | Y | N | N | N |
| Item 5. Was the data collected in a way that addressed the research issue? | Y | Y | Y | Y | Y | Y | N | Y | Y | Y | Y |
| Item 6. Has the relationship between researcher and participants been adequately considered? | Y | Y | Y | Y | N | Y | Y | Y | Y | Y | Y |
| Item 7. Have ethical issues been taken into consideration? | Y | Y | Y | Y | Y | Y | Y | Y | N | Y | N |
| Item 8. Was the data analysis sufficiently rigorous? | Y | Y | Y | Y | Y | Y | Y | Y | Y | Y | Y |
| Item 9. Is there a clear statement of findings? | N | N | N | N | N | N | N | N | Y | Y | N |
| Item 10. Was this research valuable? | Y | Y | Y | Y | Y | Y | Y | Y | Y | Y | Y |
| Overall score | 80% | 80% | 90% | 80% | 70% | 90% | 70% | 90% | 80% | 70% | 70% |

Y = Yes; N = No; C = Can’t tell
